# Definition and assessment of fever-related discomfort in pediatric literature: a systematic review

**DOI:** 10.1007/s00431-024-05753-7

**Published:** 2024-09-23

**Authors:** Gregorio P. Milani, Ilaria Alberti, Alessia Bonetti, Silvia Garattini, Antonio Corsello, Paola Marchisio, Elena Chiappini

**Affiliations:** 1 Pediatric Unit, Fondazione IRCCS Ca’ Granda Ospedale Maggiore Policlinico, Via Della Commenda 9, 20122 Milan, Italy; 2 Department of Clinical Sciences and Community Health, Università degli Studi di Milano, Milan, Italy; 3 Department of Health Sciences, Section of Paediatrics, University of Florence, Florence, Italy; 4 Paediatric Infectious Disease Unit, IRCCS Anna Meyer Children’s Hospital, Florence, Italy

**Keywords:** Fever, Management, Fever phobia, Comfort, Antipyretics

## Abstract

Guidelines advocate that the symptomatic management of fever should prioritize alleviating the child's discomfort. We investigated the definition and assessment of discomfort in febrile children within the scientific pediatric literature. A systematic review was conducted in accordance with PRISMA 2020 guidelines and preregistered on the Prospero database (CRD42023471590). Databases including PubMed, Embase, and Cochrane were searched. Studies addressing discomfort in febrile children were eligible. Out of 794 initially identified articles, 27 original studies and seven guidelines specifically used the term ‘discomfort’. Only 14 original articles provided a definition of discomfort, revealing substantial heterogeneity and no clear-cut definition. Discomfort was often assessed subjectively, predominantly through parent or self-report, and only two studies used a scoring system for assessment. The definitions varied widely, with terms such as crying, irritability, shivering and chills, pain and distress, goosebumps commonly used and evaluation of observable modifications such as facial modifications. Overall, no consensus on a single, standardized definition was available.

*Conclusions*: This systematic review shows the absence of a standardized definition and assessment of discomfort in febrile children. The findings of the present analysis might be the basis for building a consensus and developing a new tool to evaluate discomfort.

**Table:** 

**What is Known:** • *Discomfort is currently considered the main criterion to guide antipyretic administration in children with fever.* • *Despite this clear-cut recommendation, it has been questioned whether a commonly accepted understanding and assessment of this condition exists.*
**What is New:** • *This systematic review identifies a significant heterogeneity in definitions and assessment of discomfort in children with fever.* • *Both subjective parameters and observable modifications in physiological parameters should be included in a new and shared characterization of discomfort.*

**Supplementary Information:**

The online version contains supplementary material available at 10.1007/s00431-024-05753-7.

## Introduction

Fever annually affects approximately 70% of preschool-aged children, leading about 40% of these cases to seek medical care [1, 2]. Both national and international guidelines advocate that the management of fever with antipyretics, such as paracetamol or ibuprofen, should prioritize alleviating the child’s general conditions and should be prescribed only when the child presents with discomfort independently form a specific body temperature threshold [3]. Despite this clear-cut recommendation, it has been recently questioned whether a commonly accepted understanding and assessment of discomfort exists in the scientific community [4]. This issue is of paramount importance to favorite a proper management of pediatric patients with fever. For this purpose, we conducted a study to investigate existing definitions of discomfort in febrile children within literature and evaluate how this condition is assessed.

## Material and methods

### Literature search and study selection

A systematic literature review was conducted according to PRISMA 2020 guidelines. The protocol was preregistered on the Prospero database (CRD42023471590). The search was conducted on November 30, 2023, in three databases (PubMed, Embase, and Cochrane) using the following terms: (child* OR pediatric* OR perinat* OR neonat* OR newborn* OR infan* OR baby OR babies OR toddler* OR juvenil* OR adolescen*) AND (discomfort* OR comfort* AND (fever OR pyrexia OR hyperthermia OR temperature OR febrile OR feverish OR body temperature)). The detailed literature search strategy is provided in the online supplementary material. Eligible reports were original studies providing a definition of discomfort associated with fever in childhood. Studies written in languages other than English, letters, case reports, or case series with a sample size of fewer than ten subjects, and studies conducted in non-human subjects were excluded. Additionally, a search was conducted on guidelines on fever management. The search was carried out on national scientific societies or government organizations’ websites: PubMed; Australian Clinical Practice Guidelines (http://www.clinicalguidelines.gov.au/); Canadian CPG Infobase: Clinical Practice Guidelines Database (http://www.cma.ca/En/Pages/clinical-practice-guidelines.asp); Guidelines International Network (http://www.g-i-n.net/); National Guideline Clearinghouse (http://www.guideline.gov); NICE: National Institute for Health and Care Excellence (http://www.nice.org.uk); Scottish Intercollegiate Guidelines Network (SIGN) (http://www.sign.ac.uk).

### Study management, data extraction, and quality assessment

The tool Rayyan, a text mining technology to identify abstracts that are potentially most relevant for a project, allowing those abstracts to be screened first, was used to manage original articles and guidelines. Data were recorded in a predefined electronic database. From the original articles, the following data were extracted: general characteristics of the study (author, year of publication, country), study design, sample size, and definition of discomfort. For guidelines, the information collected included: authorship, publication year, country, type of guidelines and definition of discomfort.

 The STROBE guideline for observational studies and the Cochrane Risk of Bias tool for randomized controlled trials (RCTs) were used. AGREE 2 was used to evaluate the quality of guidelines.

Pairs of authors (I.A., A.S., S.G.) independently selected the articles and guidelines, extracted the data, and evaluated the study quality. In instances of discrepancies or disagreements, a collaborative approach was adopted with face-to-face discussions. If controversies persisted, a third senior author was involved (G.P.M. or E.C.)

## Results

**Fig. 1:**
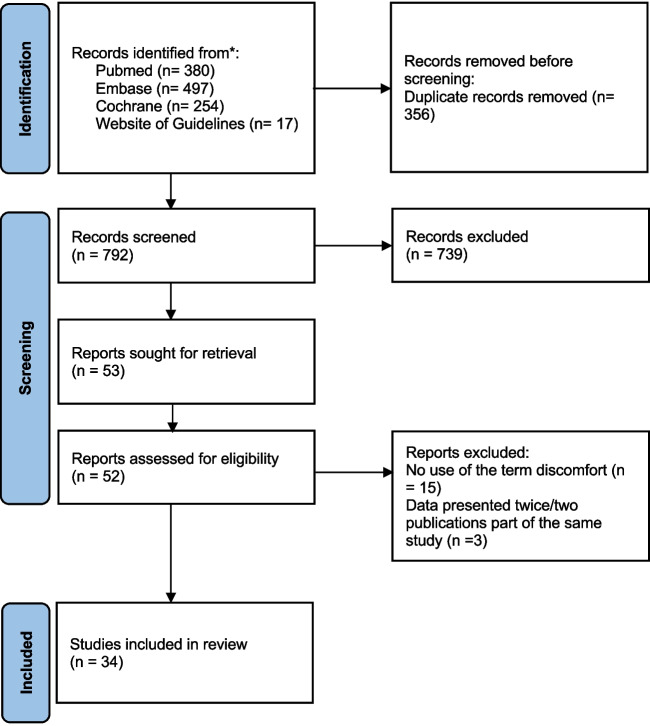
Flowchart of the literature search

A total of 794 articles (including 13 guidelines) were initially identified (Fig. 1). After the article screening, 27 original articles [5–31] and seven guidelines [32–38] that used the term “discomfort” (or “comfort”) were retrieved. Among these, 14 original articles reported a definition of discomfort [5–18]. The seven guidelines that discussed discomfort did not provide any definition of the term.

Among the original articles providing a definition of discomfort, eight were randomized clinical trials [5–12] (Table 1) and six were observational studies (four were cross-sectional studies [13, 16–18] and two were cohort studies) [14, 15] (Table 2). All the observational studies were conducted in high-income countries [13–18]. Six studies were conducted to compare the effectiveness of antipyretics, ibuprofen, or cold water sponging in the treatment of fever [5–7, 9, 11, 12]. One article established the efficacy of paracetamol and ibuprofen and their economic impact [10]. Three articles focused on analyzing fever management approaches by caregivers or healthcare providers [13, 17, 18], while one study evaluated how parents approach their children’s illness [16]. Another study analyzed the impact of paracetamol, ibuprofen, or aspirin on comfort [8]. Additionally, one study examined fever discomfort before and after paracetamol administration [14], and another compared sickness behavior and fever [15]. Children enrolled in the studies ranged in age from 6 months to 12 years.

**Table 1:** Characteristics of RCTs reporting a definition of discomfort

Name	Year	Country	Study design	Population	Study objectives	Role of discomfort in the study	Discomfort definition	Score
Agbolosu N.B. et al. [5]	1997	Malawi	RCT, data prospectively collected	80 children, aged 6 to 60 months	Compare the effects of tepid sponging and paracetamol in reducing fever in children	Discomfort used as primary outcome	Convulsions, crying, irritability, vomiting and shivering	/
Aluka T et al. [6]	2013	Nigeria	RCT, data prospectively collected	88 children from 12 to 120 months old	Compare the effectiveness of cold water sponging with that of oral paracetamol in the treatment of fever in children	Discomfort used as primary outcome	Crying, shivering, goose pimples and convulsions	/
Alves JG et al. [7]	2008	Brazil	RCT, data prospectively collected	106 children from six months to five years old with axillary temperature greater than 38°C in the emergency ward	Evaluate and compare the effectiveness of tepid sponging plus dipyrone with dipyrone alone in lowering fever in children	Discomfort used as secondary outcome	Crying, irritable or shivering	/
Autret E et al. [8]	1997	France	RCT, data prospectively collected	351 children from 6 to 24 months	To assess effectiveness and impact on children’s comfort of ibuprofen, aspirin and paracetamol	Comfort used as a primary outcome	Comfort valuated using: -Children’s reaction to pain (crying or change in facial expression) -general behaviour -child’s relief	Child’s reaction to pain: “CHEOPS” scale (child's crying: 0 not crying; 1 fretting; 2 crying or whimpering; 3 crying with sobs or screams. expression on the child's face: 0 smiling, frankly happy expression; 1 peaceful, neutral expression; 2 grimacing, frankly unhappy expression) General behavior: four level rating scale (0 as good as before the illness; 1 slightly abnormal; 2 fairly abnormal;3 very abnormal) and 100-ram horizontal visual scale (from 0 as good as before to 100 very abnormal Child’s relief: five-level rating scale (3 completely relieved; 2 fairly relieved; 1 little relieved; 0 not at all relieved; − 1 aggravated)
Chetak KB et al. [9]	2017	India	RCT, data prospectively collected	500 children under the age of 6 months to 12 years	Compare the effectiveness of tepid sponging and antipyretic drug versus antipyretic drug alone in febrile children	Discomfort used as primary outcome	Chills, goose bumps, and irritability	
Hay AD et al. [10]	2009	England	RCT, data prospectively collected	156 children Age between 6 months and 6 years	Establish the relative clinical effectiveness and cost-effectiveness of paracetamol plus ibuprofen compared with paracetamol and ibuprofen separately for time without fever and the relief of fever-associated discomfort in young children who can be managed at home	Discomfort used as primary outcome	Some pain—> distress, crying—> very distressed	/
Sharber J et al. [11]	1997	Arizona	RCT, data prospectively collected	20 children, front 5 to 68 months of age	Compare fever reduction and with acetaminophen alone and acetaminophen plus a 15-min tepid sponge bath	Discomfort used as primary outcome	Crying, shivering, and goosebumps	/
Thomas S et al. [12]	2009	India	RCT, data prospectively collected	150 children from 6 months to 12 years with fever (axillary temperature ≥ 101°F) in a tertiary care hospital	Determine which approach between tepid sponging and antipyretic drug versus only antipyretic drug is more effective in managing fever in children	Discomfort used as secondary outcome	Crying, restlessness and irritability	/

**Table 2:** Characteristics of observational studies reporting a definition of discomfort

Name	Year	Country	Study design	Population	Study objectives	Role of discomfort in the study		Discomfort definition	Score
Betz MG et al. [13]	2005	United Arab Emirates	Cross-sectional study, data prospectively collected	264 caregivers identified following the registration and triage of any child who presents with the primary concern or one of several concerns related to “fever”	Examine caregivers’ approach to fever in emergency situations	Discomfort used as primary outcome	Malaise and vomiting		/
Chiappini E et al. [14]	2023	Italy	Cohort study, data prospectively collected	172 febrile children attending Emergency Department (median age 41.7 months)	Evaluate the level of discomfort before and after administration of paracetamol	Degree of discomfort used as a primary outcome	Variations of sleep wake cycle, appetite, motor activity, mood, daily habits, facial expression (using Doria et al., 2019 study)		discomfort evaluated using items defined by Italian experts; level of pain evaluated using *Faces Pain Scale-Revised*
Corrard F et al. [15]	2017	France	Multi-center, observational study, data retrospectively collected	200 febrile children and 200 non-febrile children aged 6 months to 3 years (changes in child’s behavior was reported by parents)	Evaluate the relation between sickness behavior and fever and SB clinical components	Discomfort (and its association to SB) used as a primary outcome	Tendency to become irritated or angry, to whine or cry, change in facial expression indicative of pain. Decreased activity, lack of initiative, less liveliness, mood disorders with irritability, whimpering and greater tearfulness, reduced social interactions, less interest in the surrounding, distorted expression, decreased appetite, and disturbed sleep)		/
Lagerløv P et al. [16]	2003	Norway	Cross-sectional study, data prospectively collected	24 parents of children accessing 6 Norwegian hospitals	Evaluate how parents identify common childhood illnesses, their perspectives on the importance of fever, the influence of children’s illnesses on the daily life of the family, and how parents handle illnesses by using paracetamol	Discomfort used as primary outcome	Pain and sufferance		/
Lava SAG et al. [17]	2013	Switzerland	Cross-sectional study, data prospectively collected	322 pediatricians	Examine if there are variations in fever management across the three linguistic regions of Switzerland	Discomfort used as primary outcome	Reduced general appearance		/
Leigh S et al. [18]	2020	United Kingdom	Multi-center, observational, cross-sectional survey, data prospectively collected	98 parents of children 0–11 years and 99 healthcare providers (HCP)	Define parental and healthcare providers preferences for childhood fever management in the emergency department	HCP grade of discomfort was asked in the survey	Synonym of pain		/

Characteristics of the studies and guidelines which did not provided any definition of discomfort are given in the online supplementary material.

### Discomfort definition

None of the studies provided a clear-cut definition of discomfort. All the studies used a variety of terms, except for one study that used “discomfort” synonymously with “pain” [18] and another that equated it with a “reduced general appearance” [17]. Specifically, eight studies used the term “crying” [5–8, 10–12, 15], five used “irritability” [5, 7, 9, 12, 15], five used “shivering” or “chills” [5–7, 9, 11], three mentioned “goose pimples/bumps” [6, 9, 11], two referenced “convulsions” [5, 6], one used “malaise” [13], three used “change in facial expressions” [14, 15], one mentioned “general behavior” and “child’s relief” [8], and two referred to “vomiting” [5, 13].

**Fig. 2:**
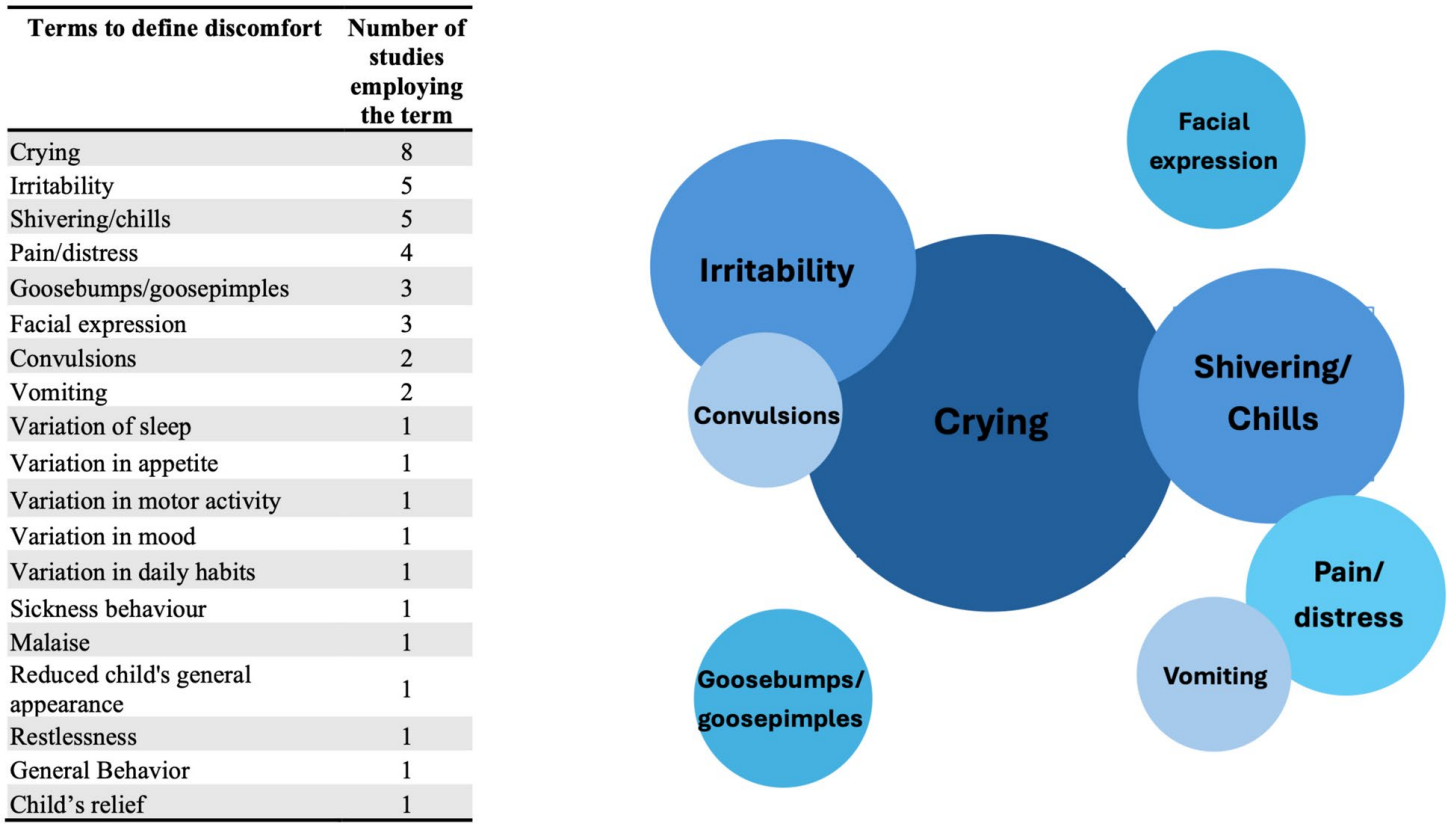
Venn diagram depicting the frequency of terms used to report discomfort. The larger the circles, the more frequently the terms to define discomfort have been used in literature. Items reported in < 2 studies were not represented by circles

One study, conducted in a high-income country, adopted a definition of discomfort from a previous study that evaluated variations in the sleep–wake cycle, motor activity, facial expressions, appetite, mood, and daily habits [14]. Another article linked discomfort to sickness behavior [15]. Additionally, four articles considered “pain” or “distress” as synonyms for discomfort (Fig. 2) [10, 15, 16, 18].

“Crying” was mentioned in seven RCTs (randomized clinical trials) conducted in Africa [5, 6], Europe [8, 10], Asia [12], North America [11], and South America [7], but it was used exclusively in only one observational study conducted in Europe [15]. “Goose pimples/bumps” and “shivering” were only mentioned in RCTs (one each in North America [11], South America [7], and Asia [9], and two in Africa[5, 6]). “Irritability” appeared in five studies (four RCTs [5, 7, 9, 12] and one observational study [15]) conducted in Africa, South America, and Asia. “Convulsions” was used to define discomfort in two RCTs conducted in Africa [5, 6], while “pain/distress”,”variations in the sleep–wake cycle”, “changes in appetite”, “variations in motor activity”, “changes in daily habits”, “sickness behavior”, and “reduced general appearance” were only used in observational studies conducted in Europe [14, 15, 17]. “Change in facial expression” was used in one RCT [8] and two observational studies conducted in Europe [14, 15].

One study utilized the Faces Pain Scale-Revised scoring system to assess discomfort, [14] while another study used a scoring system that referred to child’s reaction to pain (CHEOPS), general behavior and relief [8]. No studies evaluated vital parameters related to discomfort, and assessments were either self-reported, reported by parents, or conducted through clinical evaluation.

### Quality assessment

**Fig. 3:**
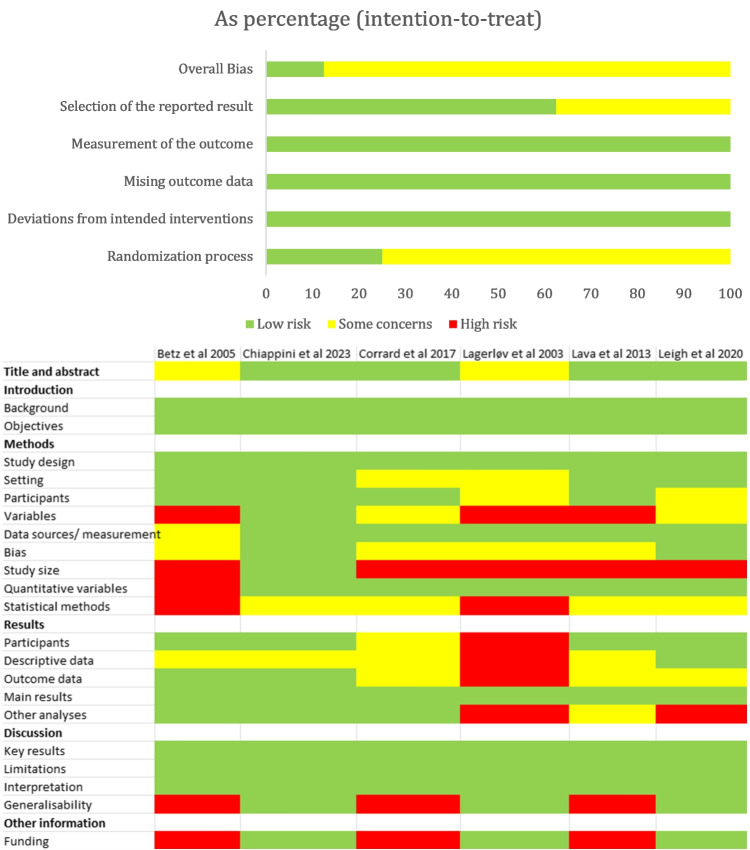
Quality assessment of RCTs (ROB) using the term discomfort and providing a definition (upper panel). Quality assessment of observational studies (Strobe) using the term discomfort and providing a definition (lower panel)

Among the seven randomized controlled trials providing a definition of discomfort (Fig. 3, upper panel), some concerns were presented in 88% of the studies [5–7, 9, 11, 12], while one exhibited a low risk of bias [10]. All the studies adhered to an intention-to-treat approach. In the case of the six observational studies providing a definition of discomfort (Fig. 3, lower panel), four exhibited a low risk of bias in the title and abstract [14, 15, 17, 18], while two raised some concerns [13, 16]. Across all six studies, there was a low risk of bias identified in the introduction [13–18]. The assessment of methods, which was categorized into study design, setting, participants, variables, data sources/measurement, bias, study size, quantitative variables, and statistical methods, generally presented some concerns. Specifically, study size showed a high risk in all studies except one, which presented a low risk of bias [14]. Results were divided into five sections: participants, descriptive data, outcome data, main results, and other analyses. One study presented a high risk of bias in four sections [16], but overall, all studies showed some concerns. Main results had a low risk of bias in all articles [13–18]. In considering the evaluation of the discussion, all articles presented a low risk of bias in key results, limitations, and interpretation [13–18]. However, three studies had a high risk of bias in generalizability [13, 15, 18], and three had a low risk [14, 16, 17]. Quality assessment of studies and guidelines which did not provide any definition of discomfort is provided in the online supplementary material.

## Discussion

This is the first systematic review investigating the presence of a shared definition and standardized assessment methods for discomfort in children with fever within the existing literature. The key findings of this analysis can be summarized as follows: (1) a minority of studies addressing this issue provide a definition of discomfort; (2) notably, pediatric guidelines on fever lack a specific definition of discomfort; (3) a lack of consensus regarding the definition of discomfort is evident in the scientific literature and it also includes guidelines on fever management; and (3) standardized methods for assessing discomfort are notably absent.

Given that fever typically holds beneficial effects for children, discomfort arising from fever can engender various challenges such as mental distress, reduced appetite, and disruptions in sleep patterns [39, 40]. Consequently, mitigating discomfort assumes importance in the management of febrile children [41, 42]. However, our review identified a marked heterogeneity in the definition of discomfort across scientific literature, independently from study design and quality. Nearly all studies employing a definition of discomfort utilized a combination of terms, often related to generic clinical manifestations or alterations in the child’s appearance and daily habits. Notably, the level of discomfort was predominantly assessed subjectively by parents or the children themselves, rendering a quantitative evaluation of discomfort challenging. Notably, only two studies incorporated a scoring system for the evaluation of discomfort.

This lack of uniformity in defining discomfort complicates the assessment and management of fever in children, potentially leading to inappropriate interventions. In a few studies, discomfort was used as a synonym of pain. While discomfort is often associated to pain, a child with fever and discomfort may present without any pain [41]. Therefore, we feel that fever, discomfort, and pain should be carefully evaluated and separately assessed.

The absence of a standardized definition of discomfort likely accounts for the dearth of standardized assessment methods in clinical practice. This finding is unexpected given the emphasis placed on treating discomfort in many guidelines, but several factors may contribute to this gap. Unlike fever, discomfort is inherently subjective, posing challenges particularly in non-verbal children such as infants. Furthermore, cultural and contextual factors in defining and assessing discomfort in febrile children might exist. We observed variations in the terminology and conceptualization of discomfort across studies conducted in different regions and settings. It is known that cultural beliefs influence conceptions on fever. Similarly, also how discomfort is perceived and expressed might vary, requiring culturally sensitive approaches to assessment and management.

The definition of discomfort might include the terms most commonly identified in this analysis such as crying, irritability, shivering and chills, pain and distress, and goosebumps. Additionally, incorporating observable alterations in physiological parameters could enhance clinical assessments (e.g., changes in facial expressions). We posit that a robust definition of discomfort should comprehensively encompass subjective experiences and objectively observable modifications in the child’s behavior. Furthermore, to facilitate widespread adoption, any new definition should be easily applicable by caregivers without specialized medical training, considering that fever management often occurs outside medical settings. To this regard, pediatric research in other fields has made several relevant improvements in recent years (e.g. introducing easy to use scales for pain assessment in children).

Prior studies have documented the prevalence of “fever phobia” among caregivers and healthcare providers, which often leads to inappropriate interventions [43–46]. Despite efforts to mitigate this phenomenon, it persists globally [44, 46, 47]. We contend that clarifying the concept of discomfort, rather than focusing solely on high body temperatures, is pivotal in altering approaches to fever management. However, the absence of a clear definition of discomfort may impede such a paradigm shift. We advocate for the creation of an international working group to provide a definition of discomfort using a standardized scientific approach, such as the Delphi process.

A standardized assessment of discomfort in clinical practice might be relevant also to evaluate the effects of pharmacological and non-pharmacological treatment of children with fever. Since most guidelines on management of children with fever highlight the importance of discomfort, future recommendations should incorporate standardized definitions of discomfort and recommend appropriate assessment strategies and interventions. Such guidelines would not only support healthcare providers in delivering optimal care but also empower parents and caregivers to effectively manage fever-related discomfort at home.

This systematic review has several limitations. The search was limited to three databases and other potential sources of articles (e.g. CINAHL) were not evaluated. All the articles considered were in English and we cannot exclude that studies in other languages providing a definition and assessment of discomfort are available. Additionally, the exclusion of narrative review may have overlooked valuable insights, although guidelines, which were expected to contain such definitions, were included. Finally, it was not possible to compare studies’ definitions is a structured way (e.g. testing if some definitions were more common in high-quality studies) due to their heterogeneity.

In conclusion, this systematic review highlights the absence of a universally shared definition and assessment of discomfort in children with fever. The data from this study might be the basis for building a consensus and developing a new tool to evaluate discomfort.

## Supplementary Information

Below is the link to the electronic supplementary material.

Supplementary file1 (DOCX 203 KB)

## Data Availability

No datasets were generated or analysed during the current study.

## Abbreviation

RCT: Randomized controlled trial
