# Supplementary material for: Definition and assessment of fever-related discomfort in pediatric literature: a systematic review
Source: Eur J Pediatr. 2024 Sep 23;183(11):4969–79. doi: 10.1007/s00431-024-05753-7 (PMC11478972; doi:10.1007/s00431-024-05753-7)
Supplement: Supplementary file 1 — Supplementary file1 (DOCX 203 KB) [file 431_2024_5753_MOESM1_ESM.docx]

**Definition and Assessment of Fever-Related Discomfort in Pediatric Literature: A Systematic Review**

**Search strategy**

**Pubmed** all fields (child* OR pediatric* OR perinat* OR neonat* OR newborn* OR infan* OR baby OR babies OR toddler* OR juvenil* OR adolescen*) AND (discomfort* OR comfort* AND (fever OR pyrexia OR hyperthermia OR temperature OR febrile OR feverish OR body temperature))

**Embase** broad search: ('child*' OR 'pediatric*' OR 'perinat*' OR 'neonat*' OR 'newborn*' OR 'infant*' OR 'baby' OR 'babies' OR 'toddler*' OR 'juvenile*' OR 'adolescent*') AND ('discomfort*' OR OR 'comfort' AND ('fever' OR 'pyrexia' OR 'hyperthermia' OR 'temperature' OR 'febrile' OR 'feverish' OR 'body temperature'))

**Web of Science** Topic search ((child* OR pediatric* OR perinat* OR neonat* OR newborn* OR infant* OR baby OR babies OR toddler* OR juvenile* OR adolescent*) AND (discomfort* OR comfort* AND (fever OR pyrexia OR hyperthermia OR temperature OR febrile OR feverish OR body temperature)))

**Supplementary table 1.** Original reports using the term discomfort (or comfort) without providing a definition

| **Name** | **Year** | **Country** | **Study design** | **Population** | **Study objectives** | **Role of discomfort in the study** | **Discomfort definition** |
| --- | --- | --- | --- | --- | --- | --- | --- |
| Chiappini E et al [26] | 2013 | Italy | Cross sectional study | 480 responses in 2009 and 300 responses in 2012 of pediatricians who participated in the 14th Italian National Congress of Practice Pediatrics in 2009 and then in the 12th National Congress of the Italian Society of Pediatric Infectious Diseases in 2012 | Evaluating the effects of the 2009 published fever guidelines on fever management | Discomfort used as primary outcome | not provided |
| Chiappini E et al [25] | 2018 | Italy | Observational, cross-sectional survey, data prospectively collected | 562 pediatricians | to evaluate the knowledge of fever phobia in pediatricians | Discomfort used as a secondary outcome | not provided |
| Deja E et al [27] | 2021 | England | Mixed methods study with data collected at the times points: (1) before, (2) during and (3) after a pilot trial | 100 children under 16 years old of age | To explore parent and staff views on the acceptability of a randomised controlled trial investigating temperature thresholds for antipyretic intervention in critically ill children with fever and infection (the FEVER trial) during a multi-phase pilot study. | Discomfort used as secondary outcome | Not provided |
| Ertmann RK et al [28] | 2012 | Denmark | Prospective longitudinal diary study | 183 caregivers of infants born in February 2001 | Assess how parents administer paracetamol during the winter, considering symptoms, medical guidance, and illness severity. | Discomfort discussed in the introduction | Not provided |
| Gupta H et al [36] | 2007 | India | Randomized clinical trial | 210 febrile children, from 6 months to 6 years | To assess the role of paracetamol in duration of fever, its effectiveness and its safety | Comfort used as a primary outcome | Not provided |
| Khaliq A et al [29] | 2019 | Pakistan | Randomized clinical trial | 100 children, mean age was 6.4±2.8 years with minimum of 2 and maximum of 13 years | To compare the antipyretic and cold sponging versus only antipyretic for treatment of fever. | Discomfort used as secondary outcome | Not provided |
| Krafft HS et al [30] | 2022 | Germany | Randomized pilot study | 24 oncology patients (children) undergoing chemotherapy | Assess the effectiveness of using heat therapy compared to the standard treatment. | Discomfort used as secondary outcome | Not provided |
| Martins M et al [31] | 2016 | Portugal | Observational cross sectional study | 270 responses from parents, 49 responses from nurses, 525 responses from doctors (228 family physicians, 291 pediatricians) | Assess the understanding of fever among parents and healthcare professionals | Discomfort used as primary outcome | Not provided |
| Milani GP et al [7] | 2023 | Italy | Cross sectional study | 121 nursing students in their final year | Examine the attitude of senior nursing students on managing fever in pediatric cases | Discomfort used as primary outcome | Not provided |
| Park Y et al [33] | 2021 | Republic of Korea | Observational study, data prospectively collected using Fever Coach app | 104337 children | to evaluate the effects between single and combination antipyretics and combination patterns of antipyretics. | discomfort used as a secondary outcome (antipyretics relieve discomfort in children with fever) | not provided |
| Peters MJ et al [34] | 2019 | United Kingdon | multi-center, three linked study:  1. qualitative study;  2.observational study;  3.randomized controlled trial. | 1.25 parents and 56 clinicians  2.1853 children admitted in PICU  3.87 children with infection and requiring mechanical ventilation | to evaluate the possibility of a RCT study in order to evaluate the clinical effectiveness and cost-effectiveness of different temperature thresholds for antipyretic use | discomfort used as a secondary outcome (cited for paracetamol use) | Not provided |
| Polidori G et al [35] | 1993 | Italy | Nonblind randomized study | 110 children age 3 to 6 years old | To compare the efficacy and tolerability of nimesulide with those of paracetamol in a nonblind randomised study that recruited children with inflammation of the upper respiratory tract and fever. | Discomfort used as primary outcome | Not provided |
| Tan E et al [37] | 2022 | New Zealand | multi center, observational, cross-sectional survey, data retrospectively collected | 602 participants (243 doctors, 353 nurses and 6 unknown) | Main outcome: evaluate the adherence to antipyretics use in children with fever.  secondary outcome: antipyretics use (for temperature reduction, for reduction of fluid intake, for febrile convulsions) and. alternating and combined antipyretic use, use of guidelines and patient information sheets. The willingness to participate in a RCT of paracetamol versus ibuprofen was also investigated. | discomfort used as a secondary outcome | not provided |

**Supplementary figure 1.** Quality assessment of RCTs (ROB) using the term discomfort without providing a definition (upper panel). Quality assessment of observational studies (Strobe) using the term discomfort without providing a definition (lower panel).

**Supplementary Table 2.** Quality assessment of guidelines (Agree II)

|  | **Scope and purpose** | **Stakeholder involvement** | **Rigour of development** | **Clarity of presentation** | **Applicability** | **Editorial Independence** | **Overall Guideline Assessment** |
| --- | --- | --- | --- | --- | --- | --- | --- |
| **American Academy of Pediatrics** [35] | **7** | **6** | **6** | **7** | **6** | **7** | **7** |
| **Italian Society of Pediatrics** [42] | **7** | **7** | **6** | **7** | **7** | **6** | **7** |
| **NICE guidelines** [4] | **7** | **5** | **6** | **6** | **7** | **6** | **6** |
| **Polish Society of Pediatric Pneumology, Polish Pediatric Society, Polish Society of Family Medicine, Polish Society of Clinical Pharmacology and Therapy** [37] | **6** | **5** | **4** | **5** | **5** | **5** | **5** |
| **Perth Children's Hospital** [39] | **6** | **6** | **4** | **4** | **5** | **5** | **5** |
| **Queensland Emergency Care Children Working Group** [41] | **7** | **7** | **5** | **6** | **7** | **5** | **6** |
| **WHO-Recommendations for management of common childhood conditions** [43] | **7** | **7** | **7** | **7** | **7** | **7** | **7** |
